# Decreased triiodothyronine (T3) as a predictor for prolonged mechanical ventilation in critically ill patients with cardiac surgery

**DOI:** 10.1186/s12871-022-01608-6

**Published:** 2022-03-09

**Authors:** Xiao Shen, Jiakui Sun, Liang Hong, Xiaochun Song, Cui Zhang, Ying Liu, Han Liu, Guojian Li, Xinwei Mu

**Affiliations:** 1 Department of Critical Care Medicine, Nanjing First Hospital, Nanjing Medical University, No. 68 Changle Road, Nanjing, 210006 People’s Republic of China; 2 Department of Orthopedics, Nanjing Yuhua Hospital, Nanjing, 210006 People’s Republic of China

**Keywords:** Prolonged mechanical ventilation, Triiodothyronine, Non-thyroidal illness syndrome, Cardiac surgery

## Abstract

**Background:**

This study aimed to examine the correlation between thyroid hormone and prolonged mechanical ventilation (MV) in adult critically ill patients having undergone cardiac surgery.

**Methods:**

The present study refers to a retrospective, cohort study conducted at Nanjing First Hospital from March 2019 to December 2020. Patients receiving cardiac surgery and admitting to the Cardiovascular Intensive Care Unit (CVICU) in the study period were screened for potential inclusion. Demographic information, thyroid hormone and other laboratory measurements and outcome variables were recorded for analysis. Prolonged MV was defined as the duration of MV after cardiac surgery longer than 5 days. Thyroid hormones were assessed for the prognostic significance for prolonged MV.

**Results:**

One thousand eight hundred ninety-six patients who underwent cardiac surgery were screened for potential enrollment. Overall, 118 patients were included and analyzed in this study. Patients fell to the control (*n* = 64) and the prolonged MV group (*n* = 54) by complying with the duration of MV after cardiac surgery. The median value of total triiodothyronine (TT3) and free triiodothyronine (FT3) were 1.03 nmol/L and 3.52 pmol/L in the prolonged MV group before cardiac surgery, significantly lower than 1.23 nmol/L (*P* = 0.005) and 3.87 pmol/L, respectively in control (*P* = 0.038). Multivariate logistic regression analysis indicated that TT3 before surgery (pre-op TT3) had an excellent prognostic significance for prolonged MV (OR: 0.049, *P* = 0.012).

**Conclusions:**

This study concluded that decreased triiodothyronine (T3) could be common in cardiac patients with prolonged MV, and it would be further reduced after patients undergo cardiac surgery. Besides, decreased T3 before surgery could act as an effective predictor for prolonged MV after cardiac surgery.

## Introduction

Low triiodothyronine (T3) syndrome, i.e., non-thyroidal illness syndrome (NTIS), has been the most common abnormality of thyroid hormone in critically ill hospitalized patients [1]. Physiologically, reduced serum T3 levels refer to the initial response of the body to acute stress to fight against catabolism [2]. However, in the condition of critical illness, such as heart failure, severe sepsis and trauma, the normal response of the hypothalamus-pituitary-thyroid (HPT) axis can alter and contribute to a decrease in T3 and thyroid-stimulating hormone (TSH), characterized as NTIS [3]. Correlations between NTIS and disease severity and prognosis in critically ill patients of the Intensive Care Unit (ICU) have been well studied. A study by Bello et al. compared the duration of mechanical ventilation (MV) and mortality between NTIS patients and non-NTIS patients in 264 patients of general ICU [4]. Results revealed a significant increase in mortality and duration of MV in the NTIS patients, indicating that NTIS might be a risk factor for critically ill patients with MV. The consistent correlation was also shown in critically ill patients with sepsis. A study conducted by Padhi et al. assessed the relationship between NTIS and mortality in 360 medical ICU patients with sepsis and found a significantly lower level of T3 in the non-survivors, indicating that low T3 might be a predictor for mortality [5].

The impact of NTIS on patients with cardiovascular diseases has also been studied in the literature. Thyroid hormone can maintain the stability of hemodynamics by influencing myocardial contractility, heart rate, myocardial oxygen consumption and peripheral vascular resistance. Changes in thyroid function usually occur after myocardial ischemia, congestive heart failure, or cardiopulmonary bypass (CPB). The NTIS has been demonstrated to be common in adult and pediatric patients having undergone cardiac surgery. Existing studies indicated that reductions in T3 were observed in patients after cardiac surgery with or without CPB [1]. Moreover, low T3 suggested as a strong predictor for mortality in heart disease patients [6]. Whereas most studies were conducted in pediatric patients, studies in adult patients having undergone cardiac surgery were relatively insufficient.

The effects of thyroid hormone on the respiratory system are also significant. Decreased thyroid hormone level could affect the ventilation function and the reaction of the respiratory center to hypoxia and hypercapnia, leading to sleep apnea or even respiratory arrest. Bello’s study showed that the duration of MV in patients with NTIS was significantly prolonged, suggesting that NTIS has a specific impact on the respiratory system of the MV patients [4]. However, there are no relevant studies on the effect of NTIS on MV patients after cardiac surgery. Therefore, our study intended to examine the correlation between thyroid hormone and prolonged mechanical ventilation (MV) in adult critically ill patients having undergone cardiac surgery.

## Material and methods

### Study design

The current study is a retrospective, cohort study that was conducted at Cardiovascular Intensive Care Unit (CVICU) of Nanjing First Hospital, an urban, tertiary care, teaching Medical College Hospital in China. Patients receiving cardiac surgery and admitting to the center of the authors from March 2019 to December 2020 were screened for potential inclusion. The data of the patients were filtered and collected from the electronic medical record (EMR) databases. The study was performed based on the Declaration of Helsinki. The Institutional Ethics Committee of Nanjing First Hospital has approved all the study protocols (KY20170811–03).

The inclusion criteria of this study included: (1) adult patients with cardiac disease who had undergone cardiac surgery with CPB, (2) patients admitted to CVICU immediately after surgery, (3) patients receiving MV for over 48 h after cardiac surgery, and (4) available assessments of thyroid hormone before and 24 h after surgery. Patients with known thyroid diseases, abnormal thyroid gland on palpation or other examinations (e.g., enlarged thyroid and thyroid nodules), who were pregnant, or on hormonal therapy were excluded here.

### Data collection

Demographic information was recorded for further analyses (e.g., age, gender, EuroSCORE, Acute Physiology and Chronic Health Evaluation II (APACHE II) score, body mass index (BMI), and co-morbidities as well as thyroid hormone and other chemical data). In addition, types of operation and operation time were collected. Moreover, outcome variables (e.g., hospital mortality, time of MV, length of hospital stay and ICU stay) were recorded for further comparison. The definition of prolonged MV was the duration of MV after cardiac surgery longer than 5 days.

### Laboratory measurements

To conduct thyroid hormone analysis, TSH, free T3 (FT3), total T3 (TT3), free thyroxine (FT4) and total thyroxine (TT4) were determined from blood samples of the patients before and 24 h after cardiac surgery. Thyroid hormone was detected with Chemiluminescence immunoassay instrument (MAGLUMI 2000, Snibe Diagnostic, China). In addition, the reference values of the hospital of the authors included: TT3: 0.98–2.33 nmol/L, TT4: 62.68–150.84 nmol/L, FT3: 2.43–6.01 pmol/L, FT4: 9.00–19.00 pmol/L and TSH: 0.35–4.94mIU/L. Lactate was obtained from arterial blood gas with ICU blood-gas analyzer (NOVA CCX Blood Gas Analyzer, USA), and white blood cell (WBC) counts were determined from blood routine test performed in the central laboratory of the hospital of the authors with the use of automatic hematology analyzer (F 560, Maccura Biotechnology, China). Biochemical analysis, involving total protein, albumin, creatinine, total bilirubin, aspartate aminotransferase (AST) and aspartate aminotransferase (ALT), was conducted in the central laboratory of the hospital of the authors by applying an Aeroset analyzer (Hitachi 7180 Automatic Biochemical Analyzer, Japan). The serum level of N-terminal pro brain natriuretic peptide (NT-proBNP) was identified using rapid diagnostic cassette and Immunoquantitative analyzer (FIA8000, Getein Biotech, China).

### Statistical analysis

Patients were divided into the control group and prolonged MV group according to the duration of MV after cardiac surgery. Categorical variables were expressed as frequency plus percentage, and continuous variables were described as median plus the interquartile range (IQR). The differences between groups were compared using the student’s t-test for continuous variables with normality distribution and the Mann-Whitney U test for those without normality distribution. The differences between groups for categorical variables were compared by performing a Chi-square test.

Correlations between variables and prolonged MV were assessed by conducting logistic regression analysis. Variables showing statistical significance in the univariate logistic regression were covered in the multivariate logistic regression. Moreover, to evaluate the predictive ability of the variables for prolonged MV, a receiver operating characteristic (ROC) curve was applied. Next, univariate and multivariate linear regression analyses were conducted to assess the correlations between variables and time of MV. Likewise, in-depth multivariate linear regression only involved the variables showing statistical significance in the univariate analysis.

All the statistical analyses were performed using IBM SPSS Statistics 26.0 (IBM Analytics, USA), and *P* < 0.05 was considered with statistical significance.

## Results

**Fig. 1:**
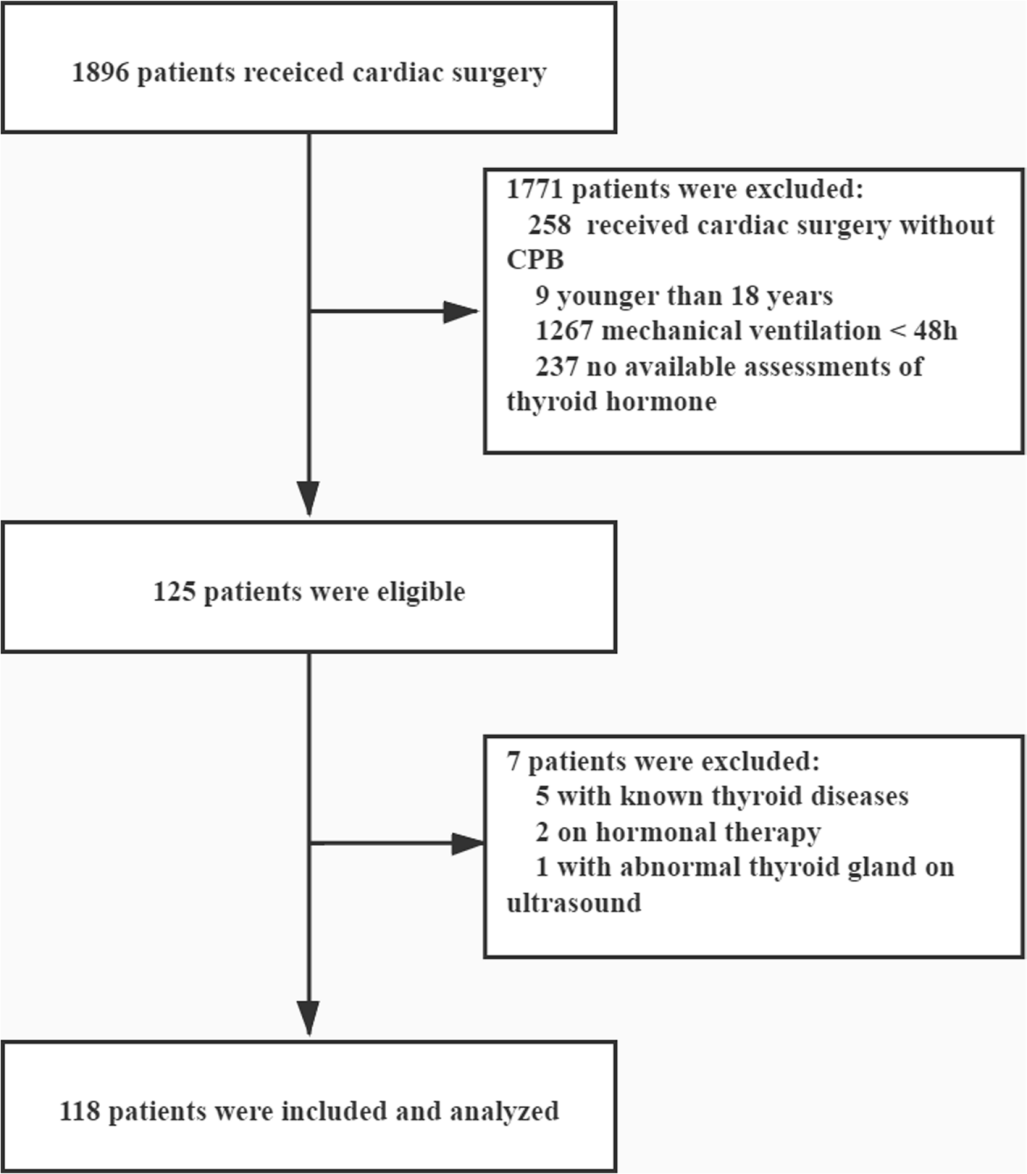
Screening of the study patients

On the whole, 1896 patients admitting to the center of the authors and having undergone cardiac surgery were screened, and 1778 patients were excluded (Fig. 1), leaving 118 patients included and analyzed in this study. Patients fell to the control (*n* = 64) and the prolonged MV group (*n* = 54) by complying with the duration of MV after cardiac surgery. Prolonged MV was defined as the time of MV longer than 5 days after cardiac surgery.

The baseline characteristics exhibited by the patients were listed in Table 1. The patients had an overall mortality of 18.6%. Specifically, 11 patients died of multiple organ failure caused by severe infection, eight patients died of malignant arrhythmia and cardiac arrest, two patients died of heart failure and multiple organ failure, and one patient died of gastrointestinal hemorrhage. Compared with the control, the prolonged MV group had a markedly longer length of hospital stay (27 days [21, 37] vs. 22 days [17, 28], *P* = 0.002) and ICU stay (12 days [8, 19] vs. 5 days [4, 10], *P* < 0.001), as well as a prolonged duration of MV (8 days [5, 10] vs. 3 days [3, 4], *P* < 0.001). No statistically significant difference was identified in other clinical parameters (e.g., gender, age, BMI, APACHE II score and euroSCORE) between the control and the prolonged MV group. The main types of cardiac surgery consisted of aortic surgery, isolated valve surgery and combined surgery of coronary artery bypass grafting (CABG) and valve surgery in prolonged MV patients. The proportion of patients having undergone aortic surgery in the prolonged MV group exceeded that in control (28 [51.8%] vs. 22 [34.4%], *P* = 0.056), though without statistical difference. The two groups had similar incidences of co-morbidities except for chronic kidney disease, higher than that of the control (4 [6.2%] vs. 0 [0%], *P* = 0.025). The operation time was comparable in the two groups of the patients, as well as the left ventricular ejection fraction (LVEF) before cardiac surgery.

**Table 1:** Baseline characteristics of the patients underwent cardiac surgery

Variable	Control group (*n* = 64)	Prolonged MV group (*n* = 54)	Total (*n* = 118)	*P* value
Male sex, n (%)	36 (56.2)	35 (64.8)	72 (61.0%)	0.344
Age, y	66 (54, 71)	66 (55, 72)	66 (54, 72)	0.906
BMI	23.9 (21.1, 26.9)	24.8 (21.0, 27.6)	24.1 (21.1, 27.6)	0.774
APACHE II score	15 (13, 18)	15 (12, 18)	15 (12, 18)	0.590
EuroSCORE	7 (5, 8)	6 (5, 8)	7 (5, 8)	0.930
Length of hospital stay, d	22 (17, 28)	27 (21, 37)	25 (19, 32)	0.002
Length of ICU stay, d	5 (4, 10)	12 (8, 19)	8 (5, 13)	< 0.001
Time of mechanical ventilation, d	3 (3, 4)	8 (5, 10)	4 (3, 7)	< 0.001
Mortality, n (%)	11 (17.2)	10 (18.5)	22 (18.6%)	0.851
Type of operation, n (%)				
Isolated valve surgery	16 (25.0)	15 (27.8)	31 (26.3)	0.733
Isolated CABG	9 (14.1)	3 (5.6)	12 (10.2)	0.223
Combined CABG and valve surgery	13 (20.3)	6 (11.1)	19 (16.1)	0.175
Aortic surgery	22 (34.4)	28 (51.8)	50 (42.4)	0.056
Other surgery	4 (6.2)	2 (3.7)	6 (5.1)	0.836
Co-morbidities, n (%)				
Coronary heart disease	28 (43.8)	15 (27.8)	42 (35.6)	0.072
Hypertension	38 (59.4)	34 (63.0)	73 (61.9)	0.691
Diabetes mellitus	14 (21.9)	9 (16.7)	23 (19.5)	0.477
Arterial fibrillation	15 (23.4)	6 (11.1)	20 (16.9)	0.081
Stroke	7 (10.9)	3 (5.6)	10 (8.5)	0.475
Chronic kidney disease	4 (6.2)	0 (0)	4 (3.4)	0.025
LVEF, %	49 (40, 62)	57 (46, 61)	53 (40, 61)	0.240
Operation time, min	350 (275, 405)	375 (290, 446)	355 (280, 440)	0.179

*MV* Mechanical ventilation, *BMI* Body mass index, *APACHE II* Acute Physiology and Chronic Health Evaluation II, *ICU* Intensive Care Unit, *CABG* Coronary artery bypass grafting, *LVEF* Left ventricular ejection fraction

**Fig. 2:**
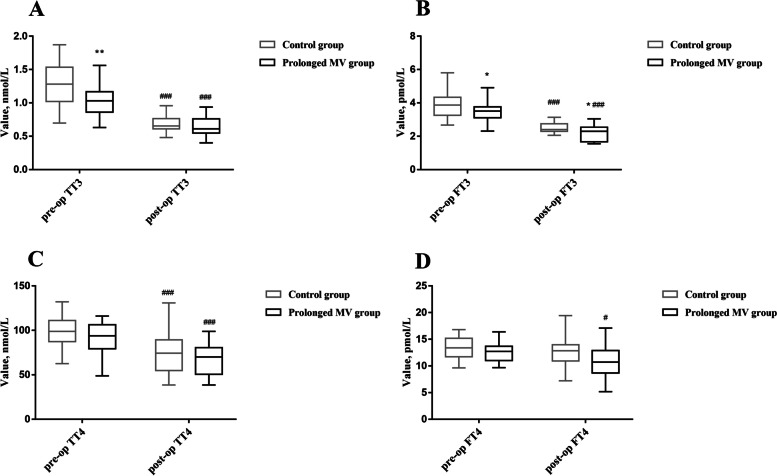
Box-and-whisker plots for thyroid hormone before and after cardiac surgery in patients of control group and prolonged MV group. **A**. Serum level of TT3 before and after cardiac surgery in the patients of the two groups. **B**. Serum level of FT3 before and after cardiac surgery in the patients of the two groups. **C**. Serum level of TT4 before and after cardiac surgery in the patients of the two groups. **D**. Serum level of FT4 before and after cardiac surgery in the patients of the two groups. *: *P* < 0.05 vs. Control group, **: *P* < 0.01 vs. Control group; #: *P* < 0.05 vs. pre-op, ##: *P* < 0.01 vs. pre-op, ###: *P* < 0.001 vs. pre-op

Table 2 listed the levels of thyroid hormones of the patients before and after cardiac surgery. The median TT3 and FT3 reached 1.03 (0.86, 1.17) nmol/L and 3.52 (3.11, 3.77) pmol/L in the prolonged MV group before cardiac surgery, significantly lower than 1.23 (1.02, 1.54) nmol/L (*P* = 0.005) and 3.87 (3.26, 4.34) pmol/L, respectively in the control (*P* = 0.038, Fig. 2). No significant differences were identified in the serum levels of TT4, FT4 and TSH before cardiac surgery in the patients of the two groups. The serum levels of thyroid hormones had an overall decrease after cardiac surgery compared with those before surgery (Fig. 2). The median FT3 after cardiac surgery was 2.30 (1.64, 2.56) pmol/L in the prolonged MV group, markedly lower than 2.40 (2.30, 2.75) pmol/L in control (*P* = 0.03, Fig. 2). However, no significant differences were reported in other indexes of thyroid hormones (e.g., TT3, TT4, FT4 and TSH) in the two groups after cardiac surgery.

**Table 2:** Serum concentration of thyroid hormones before and after cardiac surgery

Variable	Control group (*n* = 64)	Prolonged MV group (*n* = 54)	*P* value
Pre-operation			
TT3, noml/L	1.28 (1.02, 1.54)	1.03 (0.86, 1.17)	0.005
TT4, nmol/L	98.79 (87.33, 111.19)	93.93 (79.34, 106.36)	0.153
FT3, pmol/L	3.87 (3.26, 4.34)	3.52 (3.11, 3.77)	0.038
FT4, pmol/L	13.4 (11.7, 15.2)	12.72 (11.02, 13.68)	0.098
TSH, mIU/L	2.20 (1.50, 3.75)	1.68 (0.68, 3.81)	0.601
Post-operation			
TT3, nmol/L	0.66 (0.61, 0.76)	0.61 (0.54, 0.76)	0.239
TT4, nmol/L	74.38 (54.87, 89.50)	70.33 (50.46, 81.21)	0.298
FT3, pmol/L	2.40 (2.30, 2.75)	2.30 (1.64, 2.56)	0.030
FT4, pmol/L	12.86 (10.93, 13.96)	11.16 (8.66, 13.02)	0.141
TSH, mIU/L	1.23 (0.09, 2.46)	0.82 (0.18, 2.43)	0.582

*MV* Mechanical ventilation, *TT3* Total triiodothyronine, *FT3* Free triiodothyronine, *TT4* Total thyroxine, *FT4* Free thyronine, *TSH* Thyroid-stimulating hormone

Other laboratory parameters of the control and the prolonged MV group were presented in Table 3. NT-proBNP, total protein, albumin, AST, ALT, total bilirubin, creatinine, WBC as well as lactate before cardiac surgery were not different between the control and the prolonged MV group. No differences were identified in the mentioned laboratory parameters after cardiac surgery between the two groups either.

**Table 3:** Laboratory parameters of the patients before and after cardiac surgery

Variable	Control group (*n* = 64)	Prolonged MV group (*n* = 54)	*P* value
Pre-operation			
NT-proBNP, pg/ml	2143.3 (325.2, 5603.1)	2091.2 (557.4, 7506.3)	0.885
Total protein, g/L	65.8 (63.2, 69.1)	64.6 (61.0, 67.7)	0.675
Albumin, g/L	39.6 (37.1, 40.9)	38.9 (37.1, 41.6)	0.413
AST, U/L	21.0 (16.0, 30.0)	24.5 (18.0, 30.2)	0.358
ALT, U/L	17.0 (13.0, 27.0)	15.0 (10.0, 23.2)	0.267
Total bilirubin, umol/L	13.5 (9.0, 23.9)	12.3 (9.4, 18.2)	0.273
Creatinine, umol/L	76.5 (62.6, 92.6)	77.0 (64.9, 101.0)	0.527
WBC, ×10^9/L	6.8 (5.0, 8.8)	7.4 (6.2, 11.8)	0.055
Lac, mmol/L	1.4 (0.9, 2.2)	1.3 (0.8, 2.3)	0.426
Post-operation			
Total protein	49.0 (46.5, 54.3)	50.2 (44.7, 55.3)	0.892
Albumin	33.7 (31.8, 36.9)	33.1 (30.6, 37.1)	0.944
AST	63.0 (52.0, 99.0)	43.0 (36.5, 66.0)	0.620
ALT	20.0 (12.0, 30.0)	20.5 (12.2, 28.8)	0.351
Total bilirubin	18.8 (10.8, 29.6)	17.0 (13.4, 29.6)	0.472
Creatinine	85.5 (65.0, 110.4)	81.7 (65.9, 117.1)	0.426
WBC	10.4 (7.7, 16.7)	11.6 (7.7, 15.2)	0.465
PCT	1.6 (0.1, 7.1)	0.7 (0.2, 1.9)	0.230
Lac	2.8 (1.7, 5.5)	3.3 (1.6, 6.0)	0.393

*MV* Mechanical ventilation, *NT-proBNP* N-terminal pro brain natriuretic peptide, *AST* Aspartate aminotransferase, *ALT* Aspartate aminotransferase, *WBC* White blood cell, *PCT* Procalcitonin, *Lac* Lactate

Subsequently, the prognostic significance of the respective variable for prolonged MV was determined by logistic regression analysis. Univariate logistic regression indicated the correlations between prolonged MV and TT3 (pre-op TT3, Odds Ratio [OR]: 0.045, 95% confidence interval [CI]: 0.004–0.470, *P* = 0.010) and FT3 (pre-op FT3, OR: 0.401, 95% CI: 0.163–0.987, *P* = 0.047) before cardiac surgery (Table 4). According to in-depth multiple stepwise logistic regression analysis by adopting the mentioned variables, significant correlations were found between prolonged MV and pre-op TT3 (OR: 0.049, 95% CI: 0.005–0.523, *P* = 0.012, Table 5). The area under the ROC curve (AUROC) reached 0.73 (95% CI: 0.593–0.868, *P* = 0.006) for pre-op TT3, indicating high prognostic significance for prolonged MV. The specificity and sensitivity of pre-op TT3 for prolonged MV reached 84.2% and 60.6%, respectively, with a cut-off value of 1.255 nmol/L.

**Table 4:** Logistic regression for baseline and laboratory variables to predict prolonged MV

Variable	OR	95% CI	*P* value
Male sex	1.382	0.653–2.922	0.398
Age	0.998	0.970–1.028	0.905
BMI	1.012	0.933–1.097	0.772
APACHE II score	1.020	0.949–1.096	0.587
EuroSCORE	1.007	0.871–1.164	0.930
LVEF	10.311	0.210–505.667	0.240
Operation time	1.002	0.999–1.006	0.180
Pre-operation			
NT-proBNP	1.000	1.000–1.000	0.882
Total protein	0.981	0.901–1.070	0.671
Albumin	1.030	0.958–1.107	0.425
AST	1.001	0.998–1.004	0.474
ALT	0.984	0.953–1.015	0.312
Total bilirubin	0.973	0.927–1.022	0.276
Creatinine	0.998	0.993–1.004	0.529
WBC	1.118	0.999–1.252	0.053
Lac	0.881	0.636–1.219	0.443
TT3	0.045	0.004–0.470	0.010
TT4	0.975	0.942–1.010	0.156
FT3	0.401	0.163–0.987	0.047
FT4	0.763	0.551–1.055	0.102
TSH	0.931	0.716–1.211	0.594
Post-operation			
Total protein	1.003	0.957–1.052	0.891
Albumin	0.997	0.912–1.089	0.943
AST	1.001	0.998–1.003	0.631
ALT	1.001	0.999–1.003	0.379
Total bilirubin	0.991	0.968–1.015	0.470
Creatinine	0.998	0.992–1.003	0.433
WBC	0.977	0.917–1.041	0.473
PCT	0.925	0.791–1.080	0.323
Lac	1.056	0.932–1.196	0.392
TT3	0.047	0.000–7.350	0.236
TT4	0.981	0.947–1.017	0.295
FT3	0.172	0.028–1.043	0.056
FT4	0.829	0.643–1.068	0.147
TSH	1.032	0.918–1.161	0.595

*MV* Mechanical ventilation, *OR* Odds ratio, *CI* Confidence interval, *BMI* Body mass index, *APACHE II* Acute Physiology and Chronic Health Evaluation II, *LVEF* Left ventricular ejection fraction, *NT-proBNP* N-terminal pro brain natriuretic peptide, *AST* Aspartate aminotransferase, *ALT* Aspartate aminotransferase, *WBC* White blood cell, *Lac* Lactate, *TT3* Total triiodothyronine, *FT3* Free triiodothyronine, *TT4* Total thyroxine, *FT4* Free thyronine, *TSH* Thyroid-stimulating hormone, *PCT* Procalcitonin

**Table 5:** Multivariate stepwise logistic regression and AUROC for baseline and laboratory variables to predict prolonged MV

	OR (95%CI)	*P* value	AUROC (95%CI)	*P* value
Pre-op TT3	0.049 (0.005–0.523)	0.012	0.730 (0.593–0.868)	0.006

*AUROC* The area under the receiver operating characteristic curve, *OR* Odds ratio, *CT* Confidence interval, *MV* Mechanical ventilation, *pre-op TT3* Total triiodothyronine before operation

In addition, the correlations between laboratory parameters and duration of MV were also assessed. As revealed from the univariate linear regression models, LVEF (OR: 8.988, 95% CI: 0.231 to 17.745, *P* = 0.044), pre-op TT3 (OR: -3.674, 95% CI: − 6.332 to − 1.017, *P* = 0.008), FT4 after surgery (Post-op FT4, OR: -0.652, 95% CI: − 1.270 to − 0.035, *P* = 0.039) were significantly correlated with the duration of MV (Table 6). After the further multivariate analysis, as expected, LVEF (OR: -13.074, 95% CI: − 20.489 to − 5.659, *P* = 0.028), pre-op TT3 (OR: -7.916, 95% CI: − 10.352 to − 5.481, *P* = 0.015) and post-op FT4 (OR: -0.835, 95% CI: − 1.029 to − 0.640, *P* = 0.012) still showed independent and significant correlation with the duration of MV.

**Table 6:** Univariate and multivariate analyses of factors associated with MV time

Variable	Univariate analysis			Multivariate analysis		
	OR	95%CI	*P* value	OR	95%CI	*P* value
Age	0.019	− 0.047 to 0.085	0.572			
BMI	−0.041	−0.225 to 0.142	0.656			
APACHE II score	−0.020	−0.182 to 0.142	0.807			
EuroSCORE	−0.235	−0.562 to 0.091	0.156			
LVEF	8.988	0.231 to 17.745	0.044	−13.074	−20.489 to −5.659	0.028
Operation time	0.002	−0.006 to 0.010	0.572			
Pre-op TT3	−3.674	−6.332 to −1.017	0.008	−7.916	−10.352 to −5.481	0.015
Pre-op TT4	−0.041	−0.092 to 0.011	0.118			
Pre-op FT3	−0.694	−1.825 to 0.436	0.223			
Pre-op FT4	−0.401	−0.859 to 0.056	0.084			
Pre-op TSH	−0.061	−0.451 to 0.329	0.755			
Post-op TT3	−13.129	−26.618 to 0.361	0.056			
Post-op TT4	−0.086	−0.182 to 0.010	0.078			
Post-op FT3	−0.762	−5.178 to 3.654	0.728			
Post-op FT4	−0.652	−1.270 to − 0.035	0.039	− 0.835	−1.029 to − 0.640	0.012
Post-op TSH	0.088	−0.166 to 0.342	0.487			

*MV* Mechanical ventilation, *OR* Odds ratio, *CI* Confidence interval, *BMI* Body mass index, *APACHE II* Acute Physiology and Chronic Health Evaluation II, *LVEF* Left ventricular ejection fraction, *Pre-op TT3* Total triiodothyronine before operation, *Pre-op FT3* Free triiodothyronine before operation, *Pre-op TT4* Total thyroxine before operation, *Pre-op FT4* Free thyronine before operation, *Pre-op TSH* Thyroid-stimulating hormone before operation, *Post-op TT3* Total triiodothyronine after operation, *Post-op FT3* Free triiodothyronine after operation, *Post-op TT4* Total thyroxine after operation, *Post-op FT4* Free thyronine after operation, P*ost-op TSH* Thyroid-stimulating hormone after operation

## Discussion

We conducted this retrospective, cohort study to assess the prognostic significance of thyroid hormone for prolonged MV in critically ill patients having undergone cardiac surgery. As revealed from the results of this study, serum levels of thyroid hormone were down-regulated significantly in the cardiac patients having undergone cardiac surgery. Besides, prolonged MV patients after cardiac surgery had a markedly lower level of TT3 before surgery. Furthermore, TT3 before surgery significantly correlated with the duration of MV, acted as an effective predictor for prolonged MV in patients having undergone cardiac surgery.

NTIS, also known as euthyroid sick syndrome (ESS), refers to a common endocrine disorder occurring in severe stress states such as surgery, trauma and severe infection in critically ill patients. NTIS is traditionally recognized as a self-protection mechanism that down-regulates the overall metabolism to conserve energy under stress. With the gradual recovery of bodily injury, the symptoms of NTIS will be mitigated, so intervention will not be required. However, when the stress state or critical illness persists, NTIS will adversely affect the recovery of the body, thereby resulting in overcorrection. Hypothyroidism might be critical to various persistent symptoms identified in critically ill patients. Considerable studies confirmed the prognostic significance of NTIS for mortality in critically ill patients [5, 7–9]. Whereas the studies primarily concentrated on critically ill patients with the internal medical disease (e.g., sepsis and septic shock). The prognostic significance of NTIS or T3 in surgical critically ill patients remains unknown. In this study, a correlation was not identified between T3 and the mortality in the patients having undergone cardiac surgery, primarily attributed to the heterogeneity of the study population.

The critical primary condition, severe surgical trauma and short-term postoperative adverse events of the patients after cardiac surgery seriously limited the surgical efficacy and prognosis of patients, i.e., the major problems to be urgently solved by the clinical physicians at present. Patients with the cardiac disease suffer from long-term heart disease, resulting in a long-term stress state attributed to the disease, often complicated with NTIS even before cardiac surgery. Furthermore, the thyroid hormone level would further decrease after cardiac, thereby seriously affecting the prognosis of patients. Therefore, it is of great importance to determine the impact of NTIS on the prognosis of patients having undergone cardiac surgery and identify the population who need thyroid hormone replacement therapy.

Though numerous studies confirmed the prognostic significance of NTIS in medical critically ill patients, results from studies of patients with cardiac surgery remain controversial. Furthermore, most existing studies were conducted on pediatric patients having undergone cardiac surgery. Evidence from adult cardiac patients was insufficient. Several studies have demonstrated the beneficial effect of thyroid hormone replacement in patients who received cardiac surgery. A prospective study performed by Zhang et al. [10] revealed that the incidence of postoperative NTIS could be reduced, and the myocardial ischemia-reperfusion injury in pediatric patients could be protected by taking oral thyroid hormone 0.4 mg/kg for four consecutive days before cardiac surgery. The study by Marwali et al. [11] also confirmed the promoting effect of thyroid hormone replacement in pediatric patients who received cardiac surgery. They indicated that the incidence of postoperative low cardiac output syndrome could be down-regulated by administering of 1 mg/kg thyroid hormone every 6 h after surgery for patients undergoing CPB. In a prospective, multicenter, randomized, double-blind controlled study by Portman et al. [12], intravenously administered T3 effectively could up-regulate serum levels of FT3 in patients having undergone coronary artery bypass grafting for congenital heart disease without significant adverse effects and improve the cardiac function significantly. However, as suggested from a meta-analysis by Flores et al. [13], T3 treatment after CPB could not reduce mortality and duration of MV and ICU stay or improve cardiac function. Likewise, two studies in adult patients with CABG [14, 15] also showed no beneficial effect of thyroid hormone replacement for the prognosis. To date, no evidence-based guideline or consensus has been established to suggest on thyroid hormone replacement therapy in patients having undergone cardiac surgery. The exact population requiring thyroid hormone therapy and the clear time point for intervention may need to be verified by large-scale, multi-center and high-quality clinical studies. Our study confirmed the prognostic significance of T3 before surgery for prolonged MV after cardiac surgery in the cardiac patients that required MV for over 48 h after surgery, which might help prove the necessity of thyroid hormone therapy in critically ill cardiac patients.

Thyroid hormones are essential for maintaining normal respiratory function. Accordingly, hypothyroidism would affect the respiratory function adversely by impairing the function of the diaphragm and skeletal muscle and by reducing the response of the body to hypoxia and hypercapnia. A prospective cohort study by Yasar et al. [16] suggested NTIS as an independent risk factor for prolonged MV in patients with chronic obstructive pulmonary disease (COPD), complying with the results of our study. Another study by Datta et al. [17] confirmed the significance of hypothyroidism prolonged MV in the 140 medical ICU patients with respiratory failure. According to a case report by Kumar et al. [3], intravenous levothyroxine reversed the prolonged shock of uncertain etiology and difficulty weaning from MV in critically ill patients, thereby implying that low levels of thyroid hormone might be part of the cause for prolonged MV. Our study revealed that low T3 before cardiac surgery was an independent risk factor for prolonged MV in the adult critically ill patients with cardiac surgery, suggesting that thyroid hormone therapy might be beneficial for those cardiac patients with prolonged MV. Further prospective studies might be needed to identify the exact population that need thyroid hormone therapy in the adult patients having undergone cardiac surgery.

The pathophysiology of NTIS in cardiac patients remains unclear. Several mechanisms were assumed to participate in the development and progression of NTIS based on existing studies [2, 18]: hypothalamic-pituitary-thyroid axis abnormalities, peripheral thyroid hormone metabolism disorders, thyroid hormone-binding protein changes, the regulatory role of triiodothyronine receptor, cytokine effect and selenium deficiency. Vidart et al. performed a prospective clinal study in 67 patients with acute myocardial infarction to investigate the effect of N-acetylcysteine (NAC), an antioxidant, in protecting against NTIS [19]. Their study showed that NAC administration in the acute phase of acute myocardial infarction could prevent the decrease of T3 and shorten the length of hospital stay, indicating that oxidative stress responses might also be involved in the pathogenesis of NTIS of the cardiac patients. Those findings may help provide a new treatment direction for NTIS other than thyroid hormone replacement therapy. Unfortunately, we did not include any indexes of oxidative stress responses in the current study, owing to the limit of the retrospective study. However, we also found a higher level of WBC in the patients of the prolonged MV group than those of the control group, suggesting a potential correlation between NTIS and oxidative stress responses. Further large-scale prospective clinical studies or animal studies are required to elucidate the exact mechanism of NTIS in cardiac patients.

### Limitations

This study has several limitations. First, the patients with MV for over 48 h after cardiac surgery were only included, and considerable patients having undergone cardiac surgery were excluded. In this way, the results in this study may not be applicable to all the patients having undergone cardiac surgery. Second, since this study is a retrospective study, it might cause selection biases. Third, as impacted by the restriction of this retrospective study, the effect of thyroid hormone supplementation on the prognosis and prolonged MV was not assessed here.

## Conclusions

This study investigated the prognostic significance of T3 for prolonged MV in patients having undergone cardiac surgery. As revealed from this study, decreased T3 could be common in cardiac patients with prolonged MV before surgery, and it would be further reduced after patients undergoing cardiac surgery. Decreased T3 before surgery was an effective predictor for prolonged MV after cardiac surgery, thereby demonstrating that thyroid hormone therapy might be profitable in the mentioned patients.

## Data Availability

The datasets generated and/or analyzed during the current study are not publicly available due to the protection for the patients’ privacy but are available from the corresponding author on reasonable request.

## Abbreviations

MV: Mechanical ventilation
T3: Triiodothyronine
NTIS: Non-thyroidal illness syndrome
HPT: Hypothalamus-pituitary-thyroid
TSH: Thyroid-stimulating hormone
CPB: Cardiopulmonary bypass
CVICU: Cardiovascular Intensive Care Unit
BMI: Body mass index
APACHE II: Acute Physiology and Chronic Health Evaluation II
ICU: Intensive Care Unit
TT3: Total triiodothyronine
FT3: free triiodothyronine
TT4: Total thyroxine
FT4: Free thyroxine
WBC: White blood cell
ALT: Aspartate aminotransferase
AST: Aspartate aminotransferase
IQR: Interquartile range
ROC: Receiver operating characteristic
CABG: Coronary artery bypass grafting
LVEF: Left ventricular ejection fraction
OR: Odds Ratio
CI: Confidence interval
Pre-op TT3: Total triiodothyronine before operation
Pre-op FT3: Free triiodothyronine before operation
Pre-op TT4: Total thyroxine before operation
Pre-op FT4: Free thyronine before operation
Pre-op TSH: Thyroid-stimulating hormone before operation
Post-op TT3: Total triiodothyronine after operation
Post-op FT3: Free triiodothyronine after operation
Post-op TT4: Total thyroxine after operation
Post-op FT4: Free thyronine after operation
Post-op TSH: Thyroid-stimulating hormone after operation
AUROC: Area under the receiver operating characteristic curve
COPD: Chronic obstructive pulmonary disease
